# Evolutionary Characteristics and Expression Patterns of the *UGT* Gene Family in Epimedium from Gansu, China

**DOI:** 10.3390/cimb48040393

**Published:** 2026-04-11

**Authors:** Luna Xing, Jun Zhao, Qianwen Song, Chunlei Zheng, Qingyan Zhao, Wei Chen, Xiaowei Zhang, Xuhu Wang, Weibo Du, Songsong Lu, Xiaolei Zhou

**Affiliations:** College of Forestry, Gansu Agricultural University, Lanzhou 730070, China; 1073324120515@st.gsau.edu.cn (L.X.);

**Keywords:** *UGT* gene family, *Epimedium brevicornu*, molecular evolution, flavonoid glycosylation

## Abstract

*Epimedium brevicornu* is an important medicinal plant in China, whose main bioactive components are flavonoid glycosides. *UDP-glycosyltransferases (UGTs)* play key roles in flavonoid glycosylation and metabolic diversification. In this study, transcriptome data from four representative production regions in Gansu Province were used to systematically identify and analyze the *UGT* gene family in *E. brevicornu*. A total of 359 *UGT* members were identified, and 168 homologous genes with clear expression evidence were obtained from four geographical populations. Molecular evolutionary analysis showed that most *UGT* genes were under purifying selection, whereas *UGT2*, *UGT52*, *UGT57*, *UGT241*, *UGT269*, and *UGT271* exhibited significant signals of positive selection in specific lineages (*p* < 0.05). Protein interaction analysis indicated that many *UGT* proteins were closely associated with key enzymes involved in flavonoid biosynthesis, including CHS (TT4), CHI (TT5), F3H, FLS, and DFR, suggesting their potential involvement in flavonoid metabolism. Promoter analysis further revealed a high enrichment of ERF (11,169 occurrences) and MYB (7673 occurrences) transcription factor binding sites in the upstream regions of *UGT* genes. In addition, *UGT57* and *UGT241* showed significantly higher expression levels in the QLH population. Molecular docking analysis indicated relatively strong binding affinities with quercetin, with binding energies of −7.23 kcal/mol and −4.62 kcal/mol, respectively. These results suggest that the sequence variation and differential expression of *UGT* genes may be associated with flavonoid glycosylation and ecological adaptation in Epimedium. This study provides a basis for understanding the evolutionary characteristics and expression patterns of the *UGT* gene family and offers candidate genes for future studies on flavonoid metabolism.

## 1. Introduction

The genus *Epimedium*, a member of the *Berberidaceae* family, represents a distinctive lineage of perennial herbs that has undergone substantial diversification during long-term evolution [1]. Because these plants are rich in flavonoids with well-documented pharmacological activities, *Epimedium* has attracted sustained attention in modern biomedical research. Experimental studies conducted both in vitro and in vivo have shown that its major active compounds play important roles in regulating bone metabolism, suppressing inflammation, modulating immune responses, and combating oxidative stress. As a result, research on *Epimedium* has expanded from its traditional medicinal use to potential applications in the prevention and treatment of osteoporosis, neurodegenerative disorders, cardiovascular diseases, and cancer, making it an important subject in natural product and functional food research [2,3]. Approximately 80% of the reported *Epimedium* species worldwide are endemic to China. Different species exhibit marked variation in morphology, ecological adaptation, and the accumulation of chemical constituents. Flavonoids and their glycosides are among the key indicators used to evaluate the quality of *Epimedium* medicinal materials. According to the Chinese Pharmacopoeia, the total flavonoid content (calculated as icariin) in dried materials should not be less than 5.0%, and icariin itself should account for at least 0.50%. Gansu Province is recognized as one of the major production areas in China, where *Epimedium* resources are abundant and show clear regional differences in the accumulation of active compounds [4]. Even within Gansu, populations of the same species from different locations display noticeable variation in morphology and in the content of bioactive constituents. Therefore, this study selected four representative populations from different regions of Gansu Province to investigate how regional ecological differences may influence the genetic background and the formation of bioactive compounds in *Epimedium*.

Flavonoids are major active compounds in *Epimedium*, and glycosylation is a key step that determines their structural diversity, biological activity, and stability in vivo. This reaction is catalyzed by *UDP-glycosyltransferases* (*UGTs*), which use UDP-activated sugars as donors [5] and transfer sugar moieties to specific hydroxyl groups on the flavonoid backbone. As a result, flavonoids in plants mainly accumulate in the form of glycosides [6]. Glycosylation not only enhances the water solubility and chemical stability of flavonoids but also influences their physicochemical properties and intracellular distribution, thereby affecting their biological activity and storage capacity [7]. In the model plant *Arabidopsis thaliana*, genome-wide analysis has identified more than 100 *UGT* genes [8]. These genes are classified into 14 evolutionary groups and have been shown to perform diverse functions in secondary metabolism, hormone regulation, and stress responses. Similar genome-wide studies have been conducted in crops such as *Oryza sativa* and *Zea mays*, as well as in woody species including *Camellia sinensis* and *Malus domestica* [9,10,11]. In these plants, the *UGT* gene family has been systematically characterized and demonstrated to play broad roles in secondary metabolic pathways, including flavonoid glycoside biosynthesis, and in environmental adaptation. In tea plants (*Camellia sinensis*), approximately 178 *UGT* genes have been identified at the whole-genome level. Several members have been experimentally confirmed to participate in flavonoid glycoside biosynthesis. For example, *UGT*73A17 catalyzes the formation of glycosylated products from multiple flavonoid substrates [12], and its expression is closely associated with the accumulation of flavonoid glycosides.

In the genus *Epimedium*, the genome analysis of *Epimedium pubescens* has identified a large number of *UGT* genes. Previous studies have shown that some of these *UGT*s directly catalyze the glycosylation of 8-prenylated flavonoid backbones and participate in the biosynthesis of icariin and related flavonol glycosides, thereby influencing the formation and accumulation of key bioactive compounds in *Epimedium* [8]. However, most existing studies have focused on a single species or a single population, and comparative analyses of the composition and molecular evolutionary patterns of the *UGT* gene family across different production areas and natural populations remain limited. In particular, systematic research on the *UGT* gene family in *Epimedium* from the major production regions of Gansu Province is still lacking. To address this gap, the present study examined four representative populations of *Epimedium* from different regions of Gansu Province. We conducted a comprehensive analysis of *UGT* gene family members, their phylogenetic relationships, and their molecular evolutionary characteristics. The aim was to clarify how regional ecological differences may have shaped the evolutionary diversification of the *UGT* gene family and to provide a genetic basis for understanding the characteristic quality of *Epimedium* from Gansu and for future molecular breeding efforts.

## 2. Materials and Methods

### 2.1. Materials

This study established a representative gradient sampling system in the natural distribution area of *Epimedium brevicornum* in south-central Gansu Province from July to August 2024. Plants were collected from four locations. To ensure sample consistency, all leaves were taken from fresh, mature plants at the same developmental stage. The sampling sites included Hezheng County in Linxia Prefecture (HZ, 2250 m) and Qilihe District in Lanzhou (QLH, 2416 m), which are located on the western edge of the Loess Plateau and are characterized by high elevation and relatively dry conditions. Another site was Qinzhou District in Tianshui (TS, 1598 m), situated in the hilly region of southeastern Gansu, where the terrain is gentler and the climate is relatively moderate. The fourth site was Wudu District in Longnan (WD, 1736 m), located in the Qinba Mountains, where the climate is more humid and habitats are more diverse. Together, these areas form a continuous environmental gradient from dry temperate conditions to more humid mountain climates. Sampling along such environmental gradients is commonly used in studies of plant adaptation, as it helps reveal genetic differences among populations and their responses to local environmental conditions [13,14,15].

The transcriptome data of *Epimedium ilicifolium*, *E. jinchengshanense*, *E. koreanum*, *E. pseudowushanense*, *E. sagittatum*, and *E. wushanese* were obtained from previous studies conducted by our research group. The transcriptome data of *E. brevicornu* from four locations in Gansu Province (Hezheng, Qilihe, Tianshui, and Wudu) were generated from our sequencing project [16]. The whole-genome sequence of *Epimedium pubescens* used in this study was downloaded from the National Genomics Data Center (NGDC; https://ngdc.cncb.ac.cn/, accessed on 7 April 2026) [17], a publicly accessible genomic and functional gene database. The hidden Markov model (HMM) profile of the *UGT* gene family was obtained from the Pfam database (PF00201; (http://pfam.xfam.org/, accessed on 7 April 2026) [18] for conserved domain identification. The three-dimensional structure of quercetin (CAS: 117-39-5), used for molecular docking analysis, was retrieved from the PubChem database (https://pubchem.ncbi.nlm.nih.gov/, accessed on 7 April 2026).

### 2.2. Terminology

To ensure consistent naming and avoid confusion throughout the analyses, *UGT*-related genes were defined using the following conventions. The term *UGT* genes refers broadly to homologous *UGT* genes identified within the genus *Epimedium*. These genes were primarily used for comparative analyses across species, including phylogenetic reconstruction and molecular evolutionary studies. Names with geographic prefixes, such as *HZUGT1*, *QLHUGT1*, *TSUGT1*, and *WDUGT1*, represent homologous *UGT* genes or proteins identified from transcriptome datasets of *Epimedium* brevicornu populations collected from different regions of Gansu Province. All abbreviations in the article have the following meanings: *Epimedium ilicifolium* (*EPIL*), *E. jinchengshanense* (*EPJI*), *E. koreanum* (*EPKO*), *E. pseudowushanense* (*EPPS*), *E. sagittatum* (*EPSA*), and *E. wushanese* (*EPWU*). These designations were used in subsequent analyses of gene expression patterns, selection pressure, and protein structural characteristics.

### 2.3. Identification of the UGT Gene Family

The annotated protein dataset of *Epimedium pubescens* was screened using hmmsearch in HMMER v3.4 with the conserved *UDP-glycosyltransferase* domain (PF00201) from the Pfam database as the query (E-value ≤ 1 × 10^−5^) [19]. Significant matches were extracted using Python scripts (Biopython v1.79), and sequences with incomplete domains or abnormal lengths were removed. Candidate proteins were further verified using BLASTp in NCBI BLAST+ (v2.12.0) to obtain high-confidence *UGT* proteins. These sequences were then used as queries for tBLASTn searches against the *E. pubescens* genome (E-value ≤ 1 × 10^−5^) to detect potential genes missed in the initial annotation [20,21], and the corresponding CDS sequences were retrieved. The final set of *UGT* sequences was then used for one-to-one homology searches against genomes previously assembled by our research group, including 11 samples representing seven *Epimedium* species (*E. ilicifolium*, *E. jinchengshanense*, *E. koreanum*, *E. pseudowushanense*, *E. pubescens*, *E. sagittatum*, and *E. wushanese*), as well as transcriptome datasets from four populations (Tianshui, Hezheng, Wudu, and Qilihe).

### 2.4. Phylogenetic Analysis, Conserved Motifs, and Domain Characterization

To focus on the four study regions, homologous *UGT* genes were screened using a custom Perl script based on the previously identified gene set. Genes were retained when a homolog was detected in at least one of the four populations. Using the *UGT* genes of *Epimedium pubescens* as reference sequences, a total of 168 homologous genes were obtained for subsequent analyses. Protein sequences were aligned using MEGA11 [22]. Phylogenetic relationships were reconstructed using the maximum likelihood (ML) method implemented in IQ-TREE v2.2.0 [23,24], and the phylogenetic tree was visualized and refined using the iTOL online platform (https://itol.embl.de, accessed on 7 April 2026) [25].

Conserved motifs in *UGT* proteins were predicted using the MEME online server (https://meme-suite.org/, accessed on 7 April 2026) with the number of motifs set to 10 [26]. Conserved domains were identified using the Batch Web CD-Search tool provided by the NCBI (https://www.ncbi.nlm.nih.gov/Structure/cdd/wrpsb.cgi, accessed on 7 April 2026) [27]. The distribution of motifs and domain annotations was visualized using TBtools (v2.441) [28].

### 2.5. Cis-Acting Element Analysis of UGT Promoters in Epimedium pubescens

Because only transcriptome data were available for the *Epimedium* populations from Gansu analyzed in this study, a complete genome sequence was not accessible. Therefore, the published reference genome and annotation of Epimedium pubescens were used for subsequent analyses [29]. The 2000 bp sequences upstream of the transcription start site (TSS) of *UGT* genes were extracted as promoter regions using TBtools [28]. These sequences were submitted to the PlantCARE online platform (http://bioinformatics.psb.ugent.be/webtools/plantcare/html/, accessed on 7 April 2026) to predict cis-acting regulatory elements [30].

### 2.6. Transcription Factor Prediction and Protein Interaction Analysis of UGTs in Epimedium pubescens

All promoter sequences were submitted to the PlantTFDB database (http://planttfdb.gao-lab.org/, accessed on 7 April 2026) with the *p*-value threshold set to 1 × 10^−4^ [31]. The distribution of major transcription factor families was visualized as a word cloud using OmicsSuite (v1.3.9).

For the prediction of the protein–protein interaction (PPI) network, the protein sequences were compared with the Arabidopsis thaliana protein database, and homologous genes with the highest sequence similarity and clear functional annotation were selected. Based on the STRING database (https://cn.string-db.org/, accessed on 7 April 2026), the minimum required interaction score was set to 0.400 (medium confidence) [32]. The PPI network data were imported into Cytoscape (version 3.10.4), and the degree value of each node was calculated to evaluate the relative importance of different *UGT*-related proteins in the network [33].

### 2.7. Molecular Evolutionary Analysis

To analyze the natural selection pressure acting on *UGT* genes during the evolution of different Epimedium populations, molecular evolutionary analyses were performed using the codeml program in the PAML 4.9 package [34,35]. Multiple site models were compared to evaluate selection pressure at amino acid sites and to identify sites under positive selection across the entire lineage [35]. The one-ratio and two-ratio models were used to test whether the *Epimedium* populations from the four regions, treated as foreground branches, experienced different selection pressures. The branch-site model was applied to detect amino acid sites under positive selection in specific branches, and positively selected sites with a posterior probability of >95% were identified using the Bayes empirical Bayes (BEB) method. All model comparisons were evaluated using the likelihood ratio test (LRT) (degrees of freedom df = 1–2, threshold *p* < 0.05).

### 2.8. Analysis of Physicochemical Properties of UGT Proteins

*UGT* protein sequences with the closest phylogenetic relationships were selected for analysis. The number of amino acid residues, molecular weight, theoretical isoelectric point (pI), grand average of hydropathicity (GRAVY), and instability index were predicted using the ProtParam tool on the ExPASy platform (https://web.expasy.org/protparam/, accessed on 7 April 2026). In addition, the ProtScale tool (https://web.expasy.org/protscale/, accessed on 7 April 2026) was used to analyze the hydrophilicity of *UGT* proteins at the amino acid residue level [36].

### 2.9. Differential Expression Analysis of UGT Genes

The CDS sequences of *UGT* genes were used as reference sequences and aligned with the clean reads of each sample using Bowtie2 (v2.2.9) [37]. The number of reads mapped to each gene was then counted. Gene expression levels were normalized using the FPKM method [38,39]. Differences in *UGT* gene expression among regions were evaluated using one-way ANOVA, with a significance threshold of *p* < 0.05.

### 2.10. Effects of Positively Selected Mutations in UGT57 and UGT241 on Protein Structure

*UGT* genes that were widely expressed in the four *Epimedium* populations from Gansu and showed signals of positive selection in molecular evolution analyses were screened. *UGT*57 and *UGT*241, which exhibited significant adaptive evolutionary characteristics in the Qilihe population, were selected for molecular docking analysis. The three-dimensional structures of the proteins were predicted by homology modeling using the SWISS-MODEL online platform (https://swissmodel.expasy.org/, accessed on 7 April 2026), with resolved plant *UGT* protein structures from the PDB database used as templates to construct structural models of variants from different regions. The volume and opening area of the active pockets were calculated using the ProteinPlus tool (https://proteins.plus/, accessed on 7 April 2026) to evaluate structural differences in the binding pockets [40].

Using quercetin as a representative flavonoid substrate [41], molecular docking was performed with AutoDock 4.2 under default genetic algorithm parameters. The conformation with the lowest binding energy was selected as the optimal binding mode and visualized using PyMOL (version 3.1.8) for three-dimensional structure display and interaction analysis [42].

## 3. Results

### 3.1. The Identification and Phylogenetic Analysis of the UGT Gene Family

**Figure 1:**
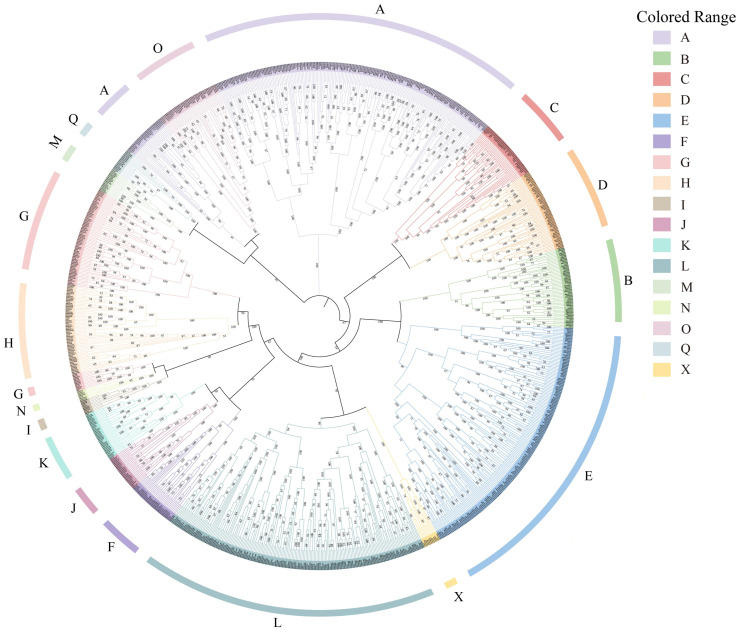
Phylogenetic reconstruction of the *UGT* gene family.

Based on genome-wide screening and phylogenetic analysis, 359 *UGT* genes were identified in *Epimedium pubescens*. From these, 168 homologous sequences were selected for detailed analysis in the four focal populations. The identification results for these 168 *UGT* genes in the genome and transcriptome datasets are provided in Supplementary Table S1. The number of matched *UGT* genes varied among species and populations. In the four Gansu populations, 136 *UGT* genes were detected in Tianshui, 138 in Qilihe, 142 in Hezheng, and 138 in Wudu. Among other *Epimedium* species analyzed, 87 *UGT* genes were identified in *E. zhenpingense*, 137 in *E. jinchengshanense*, 133 in *E. koreanum*, 159 in *E. pseudowushanense*, 153 in *E. pubescens*, 149 in *E. sagit-tatum*, and 146 in *E. wushanese*. The phylogenetic tree (Figure 1; detailed tree in Supplementary Material) revealed substantial sequence divergence among *UGT* members, reflected by the wide distribution of branch lengths. The overall topology suggests that the *UGT* gene family has undergone multiple gene duplication and diversification events, leading to the formation of numerous nested subclades. Branch length variation indicates differences in evolutionary rates among sequences, and most major nodes were strongly supported, with bootstrap values above 0.9. According to established classification criteria, the *UGT* genes of *E. pubescens* were grouped into 17 major evolutionary clades (A–Q)(See Table S2 for detailed groupings). Sixteen of these correspond to previously reported conserved *UGT* groups (A–Q), while two genes (*UGT5* and *UGT317*) did not cluster with any known group and were assigned to a separate clade (designated group X), suggesting potential structural or functional novelty (see Supplementary Table S2). Functional information from *Arabidopsis thaliana* indicates that group A is the largest clade and includes flavonol 3-O-glucosyltransferases. Group D contains flavonol 7-O-glucosyltransferases, and groups B and F also include *UGT*s involved in flavonol and anthocyanin glycosylation. In *Epimedium*, members of groups A, B, D, and F are likely associated with the glycosylation of prenylated flavonols.

### 3.2. Cis-Acting Elements, Protein Interactions, and Transcription Factor Analysis of UGT Genes in Epimedium pubescens

**Figure 2:**
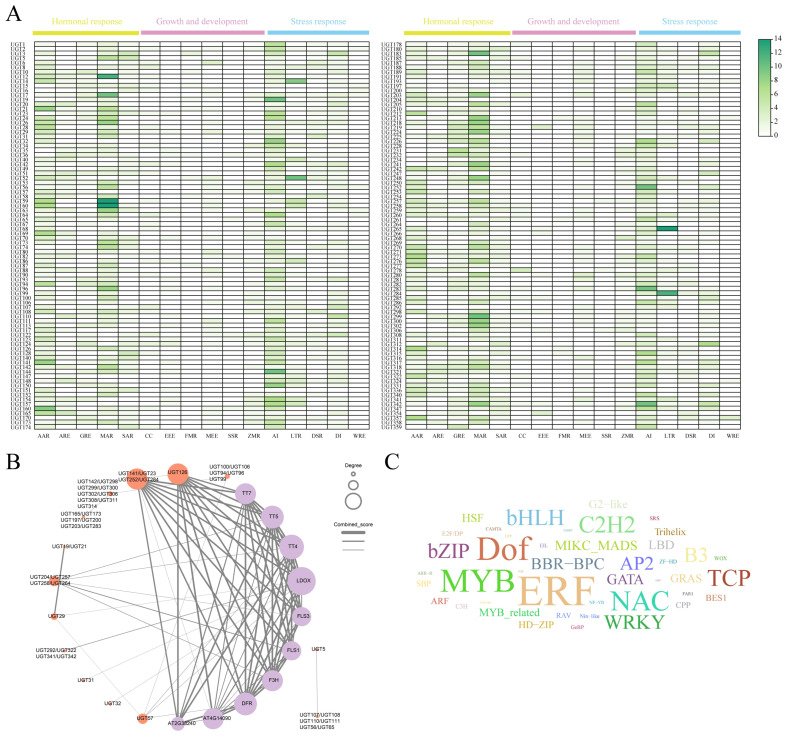
An analysis of cis-acting elements, protein–protein interactions, and transcription factors of *UGT* genes. (**A**) Cis-acting elements identified in the promoter regions of *UGT* genes. Colored blocks represent different types of regulatory elements. Yellow: Hormonal response (hormone-related elements such as ABA, auxin, and ethylene). Pink: Growth and development (elements related to cell cycle and developmental regulation). Blue: Stress response (elements related to drought, high temperature, and pathogen stress) (**B**) The predicted protein–protein interaction (PPI) network among *UGT* genes. The size of each circle is positively correlated with its degree value.The thickness of the edges represents the combined score (interaction confidence). Orange indicates UGT members, and purple indicates UGT members that can be mapped to Arabidopsis. (**C**) A word cloud of transcription factors predicted to interact with *UGT* genes. The font size is positively correlated with the number of corresponding transcription factors. (The larger the font size, the more extensive the interactions between the transcription factor and UGT genes).

The promoter regions of *UGT* genes in *Epimedium pubescens* contain a wide range of cis-acting elements related to growth and development, hormone responses, and stress adaptation.As shown in Figure 2 These elements are unevenly distributed across different genes. Light-responsive elements are the most abundant and are present in nearly all members, suggesting that many *UGT* genes may be associated with light-regulated processes. Among hormone-related elements, abscisic acid (AAR) and methyl jasmonate (MAR) response elements are the most common. Low-temperature response elements are the predominant stress-related elements. Notably, MAR and AAR elements are highly concentrated in several genes, and some members show clear enrichment of these motifs. These patterns indicate that *UGT* genes in *E. pubescens* are likely influenced by hormone signaling and may contribute to plant growth regulation and stress tolerance.

To further explore potential functional relationships, protein interaction analysis was performed for 168 *UGT* members using a homologous comparison with *Arabidopsis thaliana* (Supplementary Table S3). A total of 47 *UGT* proteins were successfully mapped to *Arabidopsis homologs*. Based on these mappings, 10 additional interacting proteins were incorporated to construct an interaction network. Most of these additional proteins are key enzymes or regulators involved in flavonoid biosynthesis, including FLS1/FLS3, DFR, F3H, and the core pathway genes TT4, TT5, and TT7. This result indicates a strong functional association between the *UGT* family in *E. pubescens* and flavonoid metabolism. Within the network, node connectivity varies markedly. TT4, TT5, TT7, FLS, DFR, and F3H display high degree values and occupy central positions, suggesting that they may play pivotal roles in *UGT*-mediated flavonoid modification and metabolic regulation. In addition, transcription factor binding site prediction based on *UGT* promoter sequences identified 73,889 potential binding sites, representing more than 40 plant transcription factor families. The distribution of these families varies substantially. The *ERF* family shows the highest frequency (11,169 sites), followed by MYB (7673), Dof (5682), NAC (4884), C2H2 (4254), and bHLH (3983). The enrichment of these transcription factor families suggests that *UGT* gene expression in *E. pubescens* is likely regulated by multiple signaling pathways and may be closely linked to developmental processes and environmental responses.

### 3.3. Molecular Evolutionary Analysis of UGT Genes

Based site model analysis showed that the overall ω (dN/dS) values for most *UGT* genes were below 1, and no significant signal of global positive selection was detected. To further test for positively selected sites across the entire gene, the M7 (beta) and M8 (beta & ω > 1) models were compared. Only *UGT2* showed a significantly better fit under the M8 model than under M7 (χ^2^ = 10.96, df = 2, *p* ≈ 0.004), indicating the presence of sites with ω > 1. In contrast, other genes, such as *UGT52* and *UGT57*, showed no significant difference between the two models (*p* > 0.05), suggesting no evidence of positive selection at the whole-gene level. These results indicate that the *UGT* gene family has mainly evolved under purifying selection. Branch model analysis further examined lineage-specific selection. Only *UGT271* on the TS branch showed a significant improvement of the two-ratio model over the one-ratio model (χ^2^ = 8.15, df = 1, *p* ≈ 0.004), suggesting an elevated ω value in this lineage. No significant branch-level positive selection was detected for other genes, including *UGT2* and *UGT269*. To refine these findings, the branch-site model was applied to candidate lineages (Table 1). Six genes showed significant likelihood ratio test results on foreground branches and contained sites with ω > 1. *WDUGT2* (*p* = 0.0003), *WDUGT52* (*p* = 0.0004), *QLHUGT241* (*p* = 0.0015), and *WDUGT269* (*p* = 0.0226) exhibited significant positive selection on their designated foreground branches (*p* < 0.05). For *WDUGT2*, *WDUGT52*, and *QLHUGT241*, the ω value on the foreground branch reached the upper boundary (ω = 999.00), indicating strong positive selection. Bayes empirical Bayes (BEB) analysis further identified specific positively selected sites, including 358V in *WDUGT2*, 133G in *WDUGT52*, 134C in *QLHUGT57*, 465D in *QLHUGT241*, 249P and 477N in *WDUGT269*, and 227T in *TSUGT271*. Overall, although the *UGT* gene family shows a generally conserved evolutionary pattern, a small number of members have experienced positive selection in specific lineages. These lineage-specific changes may be associated with functional diversification in secondary metabolism and adaptation to environmental conditions in *Epimedium*.

### 3.4. Physicochemical Properties of UGT Proteins

The physicochemical properties and sequence similarity of *UGT* proteins showing signals of positive selection were compared among four regions of Gansu Province: Wudu (WD), Qilihe (QLH), Hezheng (HZ), and Tianshui (TS) (Table 2). The results show that these *UGT* proteins range from 446 to 504 amino acids in length, with predicted molecular weights between 50.2 and 56.8 kDa. For each gene, the protein length is identical across different regions. Most *UGT* proteins have negative GRAVY values, indicating overall hydrophilic characteristics. The GRAVY values of the same gene vary only slightly among regions, although a few genes, such as *UGT271*, display slightly positive values. Regional differences were also observed in protein stability. *UGT52* from Wudu contains a mutation and shows an instability index of 37.61, which is higher than that of the Hezheng variant (34.00). Similarly, *UGT271* from Tianshui has an instability index of 48.11 due to the presence of a mutation site, which is higher than that of the Qilihe variant. Sequence similarity analysis further revealed that the same *UGT* gene exhibits high amino acid sequence similarity among different regions, with overall similarity exceeding 95%. For some gene pairs, sequence similarity reaches 98–99%, indicating strong conservation despite regional variation.

**Figure 3:**
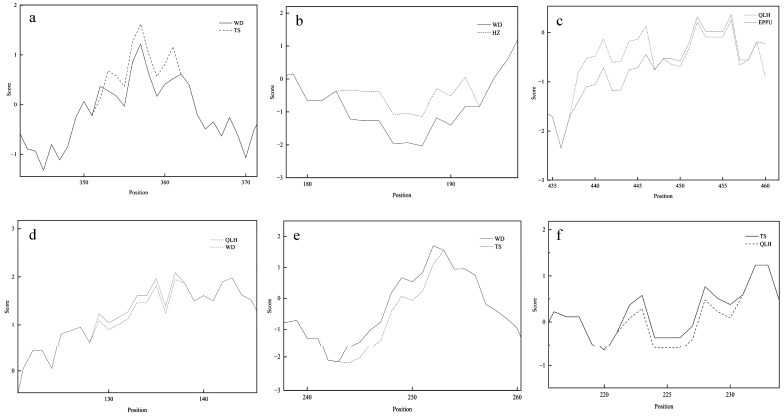
A hydropathy analysis of amino acid residues in six *UGT* genes showing signals of positive selection. The *x*-axis represents the amino acid position (Position), and the *y*-axis represents the hydrophilicity score (Score), which reflects the hydrophilicity of amino acid residues at each position. Higher scores generally indicate stronger hydrophilicity. The solid line represents the mutation region, while the dashed line represents the control region. (**a**) A comparison of *UGT2* between the Wudu and Tianshui populations. The positively selected mutation site is located at position 357 in the Wudu population. (**b**) A comparison of *UGT52* between the Wudu and Hezheng populations. The positively selected site is located at position 188 in the Wudu population. (**c**) Comparison of *UGT57* between the Qilihe population and Epimedium pubescens. The positively selected site is located at position 465 in the Qilihe population. (**d**) A comparison of *UGT241* between the Qilihe and Wudu populations. The positively selected site is located at position 134 in the Qilihe population. (**e**) A comparison of *UGT269* between the Wudu and Tianshui populations. The positively selected site is located at position 249 in the Wudu population. (**f**) A comparison of *UGT271* between the Tianshui and Qilihe populations. The positively selected site is located at position 227 in the Qilihe population.

In the hydropathy analysis, clear differences were observed among regions around the mutation sites (Supplementary Figure S1). As shown in Figure 3, in *UGT*2, the Wudu variant exhibits strong fluctuations in hydrophilicity near position 357, with peak values above +2 and troughs below −2, resulting in an overall amplitude greater than 4. In contrast, the Tianshui variant remains relatively stable in this region, showing only minor changes. For *UGT52*, at position 188, the Wudu variant shows a marked increase in hydrophilicity, with values ranging from −0.5 to +1.5. This differs substantially from the Hezheng variant, which forms a pronounced hydrophobic core in the same region (minimum value < −3; average −2.2 ± 0.4 across residues 182–190). In *UGT57*, the Qilihe variant displays strong hydropathy oscillations near position 465, with local troughs below −2.5 and peaks above +2.0. By comparison, the reference *Epimedium pubescens* sequence shows only limited variation in this region. The positively selected site of *UGT241* is located at position 134. Around this site, the Qilihe variant shows hydropathy changes exceeding 3.0 in amplitude, whereas the Wudu variant remains comparatively stable. For *UGT269*, the Wudu variant forms a distinct hydrophilic peak (>+1.5) at position 249 and a hydrophobic trough (<−2.0) at position 477. These features are not evident in the Tianshui variant. Finally, the positively selected site of *UGT271* occurs at position 227. The Tianshui variant exhibits a clear hydrophilic peak (>+2.0) at this position, while the Qilihe variant remains relatively neutral. Overall, mutations at these positively selected sites alter the local hydrophilic and hydrophobic properties of the corresponding amino acid residues. In all cases, the magnitude of hydropathy change near mutation sites is greater than in surrounding conserved regions, highlighting regional differences at key positions in *UGT* genes across ecological zones of Gansu Province.

### 3.5. Differential Gene Expression Analysis

**Figure 4:**
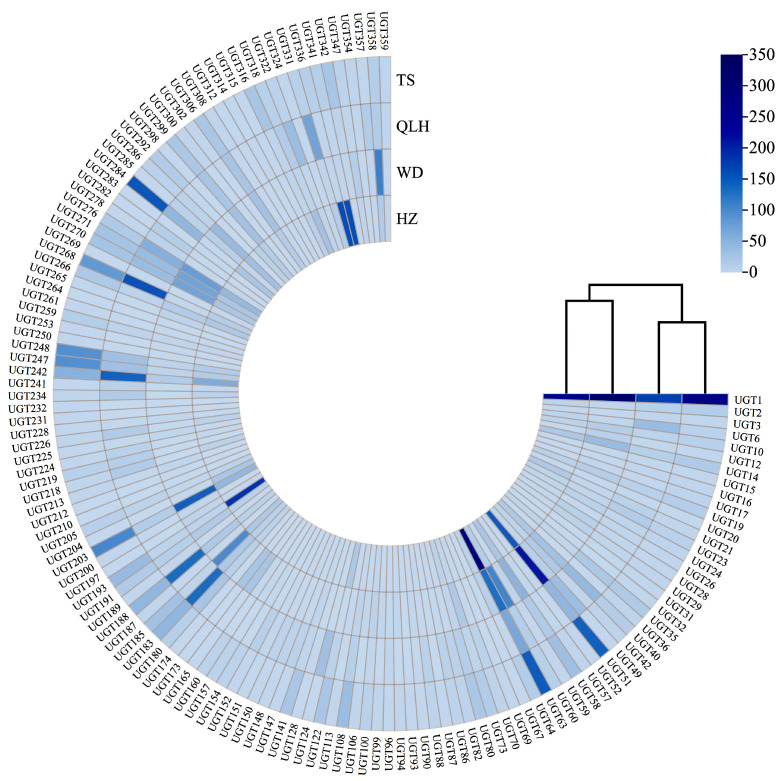
Expression levels of *UGT* genes in four different regions. The outer ring indicates the *UGT* gene names. The inner ring is divided into four sections representing the four regions: TS, QLH, WD, and HZ. Each section shows the expression level of each gene in the corresponding region.

Expression profiles of the *UGT* gene family were compared among four geographic populations in Gansu Province (Figure 4; complete data in Supplementary Table S3). Clear differences were observed in both overall expression levels and the composition of highly expressed genes among regions, indicating region-specific transcriptional patterns. In Wudu (WD), most *UGT* genes show relatively high expression levels. Genes such as *UGT51* and *UGT60* are markedly upregulated in this region, suggesting enhanced metabolic activity. In Hezheng (HZ), *UGT63*, *UGT193*, *UGT336*, and *UGT341* display prominent high expression. In contrast, Tianshui (TS) exhibits a more balanced overall expression pattern. However, key genes such as *UGT1* and *UGT284* maintain consistently high expression across all four populations, suggesting that they may perform stable and fundamental metabolic roles. Qilihe (QLH) shows the most distinctive expression pattern. Many *UGT* genes display strong regional enrichment. For example, *UGT268* reaches an expression level of 168.27 in Qilihe, compared with only 3.92 in Wudu—an increase of approximately 43-fold. Similarly, *UGT183* (142.57), *UGT242* (152.97), and *UGT49* (40.76) are also markedly upregulated in this population, forming a unique high-expression profile. Overall, these results demonstrate clear transcriptional divergence of the *UGT* gene family among different production areas in Gansu Province. This divergence suggests that regional populations may adjust glycosylation-related metabolic processes to adapt to local environmental conditions.

**Figure 5:**
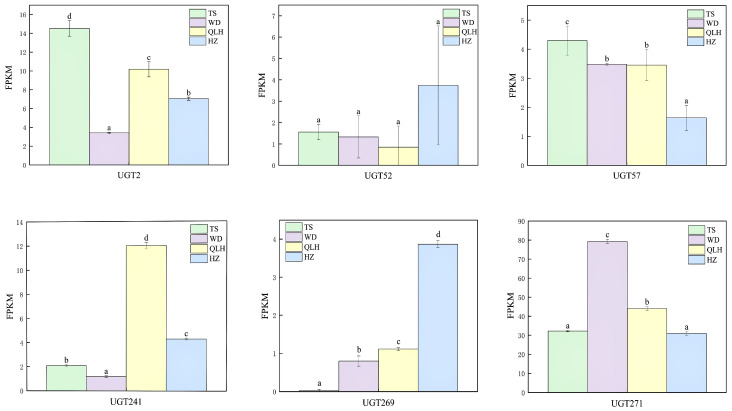
Expression levels of *UGT*2, *UGT52*, *UGT57*, *UGT241*, *UGT269*, and *UGT271* in four different regions. The *x*-axis represents different *UGT* genes, and the *y*-axis represents the expression level (FPKM). The letters above the bars (a, b, c, d) indicate statistical significance: the same letter indicates no significant difference, while different letters indicate significant differences.

Based on the FPKM expression levels of the six *UGT* genes carrying positively selected sites across four geographic populations (Figure 5), clear regional differences in expression patterns were observed. *UGT*2 shows the highest expression in Tianshui (TS) at approximately 14.7 FPKM, followed by Qilihe (QLH) at about 10.2 FPKM. Expression in Hezheng (HZ) is moderate (approximately 7.0 FPKM), while Wudu (WD) exhibits the lowest level (about 3.4 FPKM). *UGT52* displays notably higher expression in Hezheng (approximately 3.8 FPKM), exceeding the levels in Tianshui, Wudu, and Qilihe (about 1.6, 1.4, and 0.9 FPKM, respectively). Differences among the latter three regions are minimal. *UGT57* reaches its highest expression in Tianshui (approximately 4.3 FPKM), clearly exceeding the other three populations. Hezheng shows the lowest level (about 1.7 FPKM), whereas Wudu and Qilihe exhibit similar expression (both around 3.5 FPKM). *UGT271* is most highly expressed in Wudu (approximately 79.5 FPKM, *p* < 0.05), significantly higher than in the other populations. Expression levels in Tianshui and Hezheng are comparable (about 32.0 and 31.1 FPKM, respectively), while Qilihe shows an intermediate level. *UGT241* demonstrates the strongest regional specificity. Its expression in Qilihe reaches 12.1 FPKM, which is approximately 11.0-, 5.8-, and 2.8-fold higher than in Wudu, Tianshui, and Hezheng, respectively, indicating marked regional upregulation. Similarly, *UGT269* shows its highest expression in Qilihe (about 4.0 FPKM), significantly exceeding the other three populations, and it is nearly undetectable in Tianshui. Overall, the consistently elevated expression of several *UGT* genes in Qilihe highlights pronounced regional differences and suggests that local environmental conditions in this area may promote the activation of specific *UGT* genes.

### 3.6. Conserved Motif and Domain Analysis

The distribution of conserved motifs in *UGT* proteins is highly consistent with their phylogenetic classification. *UGT* members from the four regions share the same core conserved motifs, indicating that the fundamental structure of the *UGT* family is well preserved across different geographic origins. Overall, the C-terminal region of *UGT* proteins is strongly conserved throughout the gene family. In contrast, the N-terminal and central regions show clear differences in motif composition and arrangement (Supplementary Figure S2). These variations may reflect functional diversification, while the conserved regions likely maintain essential catalytic functions.

**Figure 6:**
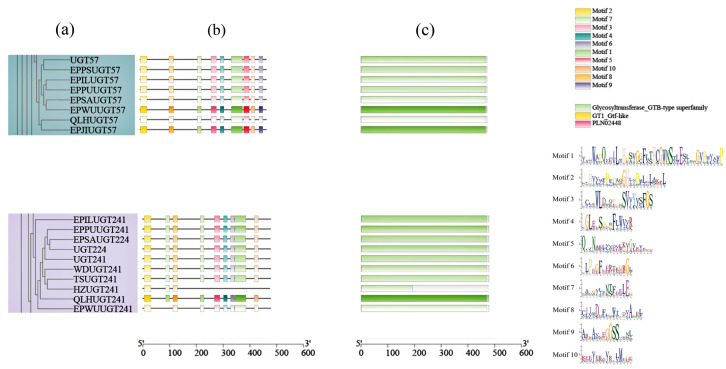
Phylogenetic tree, gene structure, and motif distribution of *UGT57* and *UGT241*. (**a**) Phylogenetic tree. (**b**) Conserved domains. (**c**) Gene structure.

To further examine structural differences in *UGT* genes showing signals of positive selection, conserved motif and domain analyses were performed for *UGT57*, *UGT241*, and their homologous members (Figure 6). Within the *UGT57* clade, motif composition is highly conserved. The ten conserved motifs predicted by MEME display nearly identical order and spatial distribution across all members, and each protein contains the typical *UGT* domain. This consistency indicates that the overall structure of the *UGT57* group has remained stable during evolution, suggesting relatively conserved functional roles. In contrast, the *UGT241* clade exhibits greater structural variability. Phylogenetic analysis shows that *UGT224* and *UGT241* cluster within the same branch. These two genes share highly similar motif composition, motif order, and domain organization, indicating a close evolutionary relationship that likely resulted from a recent gene duplication event. Although members of the *UGT241* group retain the core domain characteristic of the *UGT* superfamily, the partial loss or shortening of conserved motifs is observed in the N-terminal and central regions. Some members lack motif segments that are highly conserved in the *UGT57* clade. Importantly, these motif changes occur outside the conserved C-terminal UDP-sugar binding region. Therefore, the basic glycosyltransferase function is likely maintained, but alterations in substrate recognition or protein structural stability may have occurred.

### 3.7. Effects of Positively Selected Sites on the Protein Structures of UGT57 and UGT241

**Figure 7:**
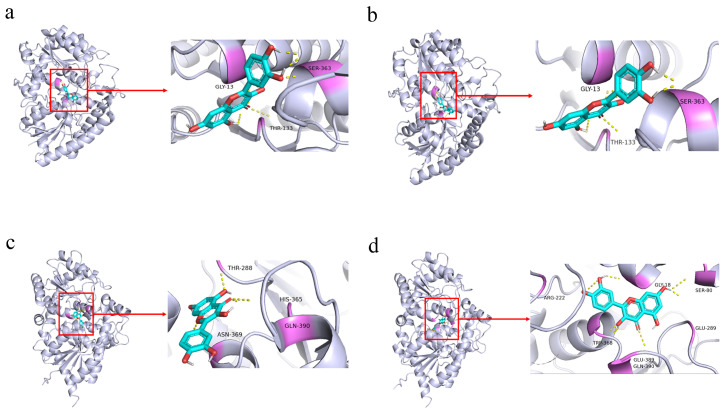
A comparison of three-dimensional protein structures and substrate-binding features of *UGT57* and *UGT241*. The part circled in red in the image is the quercetin molecule. (**a**,**b**) Protein–substrate conformations of *UGT57* from the Qilihe population and the reference *Epimedium pubescens*, respectively. (**c**,**d**) Protein–substrate conformations of *UGT241* from the Qilihe and Wudu populations, respectively. (Gray represents the overall protein structure; yellow dashed lines indicate interactions between the ligand and specific residues in the protein; purple highlights the specific amino acid residues that interact with the ligand).

Molecular docking analysis was performed to evaluate the structural effects of positively selected mutations (Figure 7). In the Qilihe (QLH) population, the amino acid substitution at position 465 of *UGT57* is not located directly within the catalytic center, whose conserved active residues include Gly13, Thr133, and Ser363. However, this mutation appears to influence the spatial arrangement around the active site. In the QLH variant, the side-chain orientations of Ser363 and Thr133—both containing hydroxyl groups—differ from those in the reference *Epimedium pubescens UGT57* (EPPU) during binding with quercetin. These conformational changes promote a more stable hydrogen bonding network. In addition, the mutation may alter the overall shape of the binding pocket, increasing its volume to 588.95 Å^3^ compared with 481.48 Å^3^ in EPPU. The calculated binding free energy between *UGT57* (QLH) and quercetin is −7.23 kcal/mol, which is lower than that of EPPU (−6.62 kcal/mol). This result suggests that the mutation enhances quercetin affinity by improving hydrogen bond interactions and structural stability.

Similarly, the Qilihe variant of *UGT241*, which carries a positively selected site, shows stronger binding to quercetin than the Wudu (WD) variant. The binding free energy of QLH-*UGT241* (−4.62 kcal/mol) is markedly lower than that of WD-*UGT241* (−2.38 kcal/mol). The QLH variant forms a smaller but deeper binding pocket (425.86 Å^3^; 13.95 Å), whereas the WD variant has a larger but shallower cavity (492.92 Å^3^; 12.64 Å). This indicates that the QLH protein adopts a more compact binding conformation. In QLH-*UGT241*, key residues such as Thr288, Asn369, and Gln390 are positioned closer to the ligand and participate in hydrogen bonding or stable polar interactions. In contrast, the corresponding residues in WD-*UGT241* are more spatially dispersed.

These structural and interaction differences are consistent with the positive selection signals identified in the branch-site analysis. Together, these results suggest that positive selection may have modified the geometry of the binding pocket and the interaction patterns of key residues in *UGT57* and *UGT241*, thereby affecting their substrate-binding properties.

## 4. Discussion

### 4.1. The Expansion of the UGT Gene Family in Epimedium and Its Relationship with Flavonoid Metabolism

The *UGT* (*UDP-glycosyltransferase*) gene family plays an important role in plant secondary metabolism. These enzymes transfer sugar groups to a wide range of small molecules, thereby influencing the structural diversity, biological activity, and stability of metabolites. In plants, the *UGT* family is typically large and phylogenetically diverse. Its expansion is generally attributed to repeated gene duplication events followed by functional diversification during long-term evolution [43].

Based on genome-wide identification in *Epimedium pubescens*, a total of 359 *UGT* family members were identified in this study. This number is substantially higher than that reported in model plants such as *Arabidopsis thaliana* and *Oryza sativa* [44]. The expansion of this gene family may be associated with the complex secondary metabolic system of medicinal plants, where extensive glycosylation reactions are required to modify diverse flavonoid compounds. Previous studies have shown that the large-scale expansion of the *UGT* family is often associated with increasing complexity in plant secondary metabolic pathways, particularly in medicinal plants rich in flavonoid glycosides [45,46]. Therefore, the extensive expansion of *UGT* genes in *Epimedium* likely provides an important genetic basis for the structural diversity and abundance of flavonoid glycosides in this genus. Using transcriptome data from four geographic populations in Gansu Province, 168 homologous *UGT* genes were further selected for detailed analysis. Although slight differences in the number of detectable *UGT* members were observed among populations, the overall composition was highly consistent. *UGT* genes belong to a superfamily characterized by a conserved glycosyltransferase domain and are classified into multiple phylogenetic groups. This pattern suggests that they have been maintained under strong purifying selection and perform essential metabolic functions. Similar observations have been reported in other plant *UGT* families, where expansion is thought to preserve core metabolic roles while also providing evolutionary flexibility for environmental adaptation and metabolic diversification [47].

### 4.2. Phylogenetic Classification and Its Relationship with Flavonoid Glycosylation

Phylogenetic analysis indicated that the *UGT* gene family in *Epimedium* can be divided into 17 major clusters (A–Q and X). These clusters reflect the evolutionary relationships among *UGT* genes and may also suggest functional differences [48]. Variations among clusters in promoter cis-elements, expression patterns, and potential interactions with other metabolic enzymes may influence substrate recognition and responses to environmental signals. Among these clusters, A, B, D, and F contain a relatively large number of *UGT* members associated with flavonoid glycosylation. Previous studies in *Arabidopsis* have shown that cluster A *UGTs* are mainly involved in flavonoid 3-O-glycosylation, whereas cluster D members are more often related to 7-O-glycosylation [8]. In this study, these clusters include more *UGT* genes in the *Epimedium* genome. This pattern is consistent with the abundant and structurally diverse flavonoid glycosides accumulated in *Epimedium*, suggesting that *UGT* genes in these clusters may play important roles in flavonoid glycosylation [49]. In addition to the clusters closely associated with flavonoid metabolism, other clusters (such as C, E, G, and H) may participate in the modification of a wider range of secondary metabolites. Previous studies have shown that some plant *UGTs* are involved in hormone metabolism, including the glycosylation of auxin and brassinosteroids, and may also function in responses to abiotic stress [50,51]. Therefore, *UGT* members in these clusters may contribute to maintaining metabolic balance and supporting environmental adaptation in *Epimedium*. Notably, the X cluster identified in this study, which includes *UGT5* and *UGT317*, does not group with any previously reported conserved *UGT* clusters and occupies an independent phylogenetic position. This result suggests that the cluster may have distinct sequence features or substrate preferences and may represent a lineage-specific group within the *Epimedium UGT* family. Its precise function requires further experimental verification.

Overall, functional differences among *UGT* clusters may result from gene duplication followed by functional divergence. Under natural selection, duplicated genes can gradually develop different expression patterns and catalytic properties [1], enabling plants to produce diverse secondary metabolites in different environmental conditions [47].

### 4.3. Cis-Acting Elements and Expression Differences

Cis-acting elements in the promoter regions of *UGT* genes form the basis for transcriptional responses to environmental signals. In this study, numerous elements related to light signaling, plant hormones, and stress responses were identified in the promoter regions of *UGT* genes in *Epimedium pubescens*. At the same time, clear differences in *UGT* gene expression were observed among different geographic populations. Light-responsive elements are widely distributed in *UGT* promoters, suggesting that light may serve as a key environmental factor regulating *UGT* expression. Since flavonoid compounds play important roles in protecting plants from ultraviolet radiation and oxidative stress, the light-mediated regulation of *UGT* genes may contribute to adaptive metabolic responses, suggesting that light may be an important external factor regulating *UGT* expression. Previous studies have shown that light conditions strongly influence the synthesis and accumulation of flavonoids [52], and glycosylation is a key step that determines their stability and storage form. In addition, *UGT* promoters are enriched with hormone-responsive elements, including ABRE (abscisic acid response), TGACG/CGTCA motifs (methyl jasmonate response), and the TCA element (salicylic acid response). These findings indicate that hormone signaling pathways likely play an important role in regulating *UGT* transcription [53]. Elements associated with abiotic stress, such as low-temperature response (LTR), drought response (MBS), and anaerobic response (ARE), were also detected. These elements provide a molecular explanation for the differences in *UGT* expression under varying ecological conditions [54,55]. *UGT*-mediated glycosylation can modify the solubility and stability of metabolites, thereby contributing to stress tolerance. Taken together, different ecological environments may influence *UGT* gene expression through promoter-mediated transcriptional regulation. This selective expression of specific *UGT* genes in distinct populations may enhance the adaptive capacity of *Epimedium* to diverse habitats.

### 4.4. Protein Interaction and Transcription Factor Analyses Reveal the Central Role of UGTs in the Flavonoid Metabolic Network

Protein interaction and transcription factor prediction analyses indicate that *UGT* genes do not function independently but are closely associated with enzymes involved in flavonoid biosynthesis, suggesting that *UGT-mediated glycosylation* represents an important downstream modification step in the flavonoid metabolic pathway. *UGT* proteins show close associations with key enzymes in the flavonoid biosynthetic pathway, including TT4 (*chalcone synthase*, CHS), TT5 (*chalcone isomerase*, CHI), F3H (*flavanone 3-hydroxylase*), FLS (*flavonol synthase*), and DFR (*dihydroflavonol 4-reductase*). These interactions suggest coordinated activity between *UGT*-mediated glycosylation and both the upstream formation of the flavonoid backbone and downstream modification steps. Promoter analysis further revealed an enrichment of binding sites for major flavonoid-related transcription factor families, including MYB, ERF, bHLH, and WRKY. Among these, MYB binding sites were the second most abundant after ERF, indicating that MYB proteins may play a central role in regulating *UGT* gene expression. Previous studies support this view. In peach (*Prunus persica*), MYB10 directly activates the promoters of UDP-glucose:flavonoid 3-O-glucosyltransferase (UFGT) and dihydroflavonol 4-reductase (DFR), thereby promoting anthocyanin accumulation [56]. In *Arabidopsis thaliana*, apple, and tea plants, *MYB–UGT* co-regulatory modules have been repeatedly shown to be closely associated with flavonoid glycoside biosynthesis [57,58]. In addition, ERF, bHLH, and WRKY transcription factors often interact with MYB proteins to form coordinated regulatory networks, such as the MBW complex, which collectively regulate flavonoid biosynthesis and subsequent glycosylation processes [59,60,61,62]. Taken together with the protein interaction and promoter prediction results, these findings support the conclusion that *UGT* genes in *Epimedium* are embedded in a complex network controlled by multiple transcription factors. Within this network, *UGT*s contribute to flavonoid metabolism not only through their catalytic activity but also through tightly coordinated transcriptional regulation. These results suggest that *UGT*-mediated glycosylation acts as an important downstream modification step in the flavonoid biosynthetic pathway.

### 4.5. Adaptive Divergence of UGT Genes in Epimedium from Gansu

Molecular evolutionary analysis showed that the overall ω (dN/dS) ratio of the *UGT* gene family was significantly lower than 1, indicating that most *UGT* genes have undergone strong purifying selection during evolution and maintain relatively conserved functions [7]. This pattern is consistent with the evolutionary trends reported for *UGT* families in other plant species and highlights their essential role in basic secondary metabolism. Despite this overall conservation, significant signals of positive selection were detected in specific lineages, including *UGT*2, *UGT52*, *UGT57*, *UGT241*, *UGT269*, and *UGT271*. In particular, the positively selected sites identified in *UGT57* and *UGT241* are mainly located in the N-terminal and central regions of the proteins. Previous structural and functional studies have shown that the C-terminal region of plant *UGT* proteins is highly conserved and primarily responsible for binding the UDP-sugar donor [5]. In contrast, the N-terminal and central regions play key roles in substrate recognition, shaping the binding pocket, and determining substrate specificity [63]. Therefore, the positively selected sites detected in *UGT57* and *UGT241* are more likely to fine-tune substrate preference and catalytic efficiency by subtly altering the local structure of the substrate-binding pocket rather than disrupting the core glycosyltransferase activity [64]. Subsequent three-dimensional structural modeling and molecular docking analyses further support this functional interpretation.

Environmental heterogeneity among different regions of Gansu Province may impose distinct selective pressures on plant metabolic genes. Qilihe is located in the high-altitude inland region of central Gansu and is characterized by large diurnal temperature fluctuations, frequent low-temperature stress, strong ultraviolet radiation, and unstable water availability. Under these environmental conditions, this study found that *UGT57* and *UGT241* in the Qilihe population not only carry amino acid substitutions driven by positive selection but also show coordinated upregulation at the transcriptional level. These molecular changes appear to optimize the spatial configuration of the substrate-binding pocket and significantly enhance the glycosylation capacity of these enzymes toward flavonoid substrates such as quercetin. Previous studies have shown that glycosylation improves the stability, solubility, and antioxidant activity of flavonoids, thereby promoting their sustained accumulation and protective function under low-temperature, high-light, and oxidative stress conditions [65]. Therefore, the positive selection signals observed in *UGT* genes from the Qilihe population likely reflect an adaptive strategy in *Epimedium*. By increasing the efficiency and stable accumulation of flavonoid glycosides, these genetic changes contribute to improved tolerance to cold, strong radiation, and multiple abiotic stresses. This pattern highlights the important role of environmental selection pressure in driving the functional divergence of *UGT* genes.

Taken together, the expansion, diversification, and adaptive evolution of *UGT* genes in Epimedium appear to be closely associated with the biosynthesis and modification of flavonoid glycosides. These results provide new insights into the molecular mechanisms underlying metabolic diversity and ecological adaptation in medicinal plants.

## 5. Conclusions

In this study, the *UGT* gene family in *Epimedium* was systematically identified, yielding 359 members—significantly more than reported in model plants—and showing notable enrichment in phylogenetic groups associated with flavonoid glycosylation. Combining transcriptome data from four geographic populations in Gansu, 168 homologous *UGT* genes with clear expression evidence were selected. Phylogenetic analysis classified these genes into 17 conserved groups, with A, B, D, and F groups being predominant, indicating a significant expansion of flavonoid glycosylation-related genes in *Epimedium*. Promoter cis-element analysis revealed that *UGT* genes are broadly regulated by light, plant hormones, and various abiotic stress-related elements, showing significant expression differences among populations. Protein–protein interaction and transcription factor predictions indicated that *UGT* proteins tightly interact with key enzymes of the flavonoid biosynthesis pathway (CHS, CHI, F3H, FLS, and DFR), and their promoters are enriched with potential binding sites for MYB, ERF, bHLH, and WRKY transcription factors, highlighting their central role in the flavonoid metabolic network. Molecular evolution analysis revealed that *UGT57* and *UGT241* experienced positive selection in specific lineages, with key sites located in substrate recognition regions, accompanied by upregulated expression in high-altitude populations, thereby enhancing their flavonoid glycosylation capacity. Collectively, these results reveal the molecular basis of the adaptive differentiation of *Epimedium UGT* genes across different Gansu regions and provide insights into the genetic foundations of high-quality medicinal traits. Furthermore, candidate genes such as *UGT57* and *UGT241* represent promising targets for functional validation and molecular breeding to improve flavonoid glycoside content and environmental adaptability.

## Figures and Tables

**Table 1:** A branch-site model analysis of *UGT2*, *UGT52*, *UGT57*, *UGT241*, *UGT269*, and *UGT271* genes in *Epimedium* based on the maximum likelihood method.

Gene	Mode	Estimates of Parameters					NP	InL	χ2	df		P	BEB
*WDUGT2*	Model A0	proportion	0.72	0.28	0.00	0.00							357 G 0.545
		background w	0.00	1.00	0.00	1.00	23	2318.45		Model A0	vs.	Model A1	358 V 0.853
		foreground w	0.00	1.00	1.00	1.00			13.15	1.00		0.0003	
	Model A1	proportion	0.73	0.27	0.00	0.00							
		background w	0.00	1.00	0.00	1.00	24	2311.88					
		foreground w	0.00	1.00	999.00	999.00							
*WDUGT52*	Model A0	proportion	0.00	0.97	0.00	0.03							188 I 0.747
		background w	1.00	1.00	1.00	1.00	11	1934.58		Model A0	vs.	Model A1	
		foreground w	1.00	1.00	1.00	1.00			12.41	1.00		0.0004	
	Model A1	proportion	0.99	0.00	0.01	0.00							
		background w	0.68	1.00	0.68	1.00	12	1928.37					
		foreground w	0.68	1.00	999.00	999.00							
*QLHUGT57*	Model A0	proportion	0.45	0.55	0.00	0.00							465 D 0.763
		background w	0.00	1.00	0.00	1.00	15	2082.38		Model A0	vs.	Model A1	
		foreground w	0.00	1.00	1.00	1.00			4.70	1.00		0.0302	
	Model A1	proportion	0.99	0.00	0.00	0.00							
		background w	0.51	1.00	0.51	1.00	16	2080.03					
		foreground w	0.51	1.00	110.87	110.87							
*QLHUGT241*	Model A0	proportion	0.56	0.44	0.00	0.00							134 C 0.884
		background w	0.00	1.00	0.00	1.00	15	2072.80		Model A0	vs.	Model A1	
		foreground w	0.00	1.00	1.00	1.00			10.13	1.00		0.0015	
	Model A1	proportion	0.99	0.00	0.00	0.00							
		background w	0.42	1.00	0.42	1.00	16	2067.73					
		foreground w	0.42	1.00	999.00	999.00							
*WDUGT269*	Model A0	proportion	0.72	0.28	0.00	0.00							249 P 0.505
		background w	0.00	1.00	0.00	1.00	19	2662.42		Model A0	vs.	Model A1	477 N 0.840
		foreground w	0.00	1.00	1.00	1.00			5.20	1.00		0.0226	
	Model A1	proportion	0.72	0.28	0.00	0.00							
		background w	0.00	1.00	0.00	1.00	20	2659.82					
		foreground w	0.00	1.00	155.11	155.11							
*TSUGT271*	Model A0	proportion	0.00	0.00	0.60	0.40							227 T 0.964
		background w	0.00	1.00	0.00	1.00	19	2558.08		Model A0	vs.	Model A1	
		foreground w	0.00	1.00	1.00	1.00			4.67	1.00		0.0307	
	Model A1	proportion	0.00	0.00	0.60	0.40							
		background w	0.00	1.00	0.00	1.00	20	2555.74					
		foreground w	0.00	1.00	434.56	434.56							

**Table 2:** Physicochemical properties of proteins.

Polypeptide Chain	Number of Amino Acid	Molecular Weight	Isoelectric Point	Gravy	Instability Index
*WDUGT2*	446	50,262.54	5.44	−0.134	40.39
*TSUGT2*	446	50,209.47	5.36	−0.117	39.79
*WDUGT52*	464	51,894.40	5.35	−0.178	37.61
*HZUGT52*	464	51,808.29	5.35	−0.166	34
*QLHUGT57*	465	52,538.09	4.99	−0.201	39.86
*EPPUUGT57*	465	52,586.09	4.99	−0.216	39.38
*QLHUGT241*	476	53,076.25	5.45	−0.059	41.17
*WDUGT241*	476	53,066.23	5.45	−0.062	41.17
*WDUGT269*	504	56,842.25	6.44	−0.120	46.28
*TSUGT269*	504	56,828.14	6.34	−0.141	47.42
*TSUGT271*	457	50,681.48	5.73	0.079	48.11
*QLHUGT271*	457	50,547.08	5.34	0.05	45.4

## Data Availability

The dataset is available on request from the authors; the raw data supporting the conclusions of this article will be made available by the authors on request.

## Supplementary Materials

The following supporting information can be downloaded at https://www.mdpi.com/article/10.3390/cimb48040393/s1.
