# Supplementary figures and images for: Evolutionary Characteristics and Expression Patterns of the UGT Gene Family in Epimedium from Gansu, China
Source: Curr Issues Mol Biol. 2026 Apr 11;48(4):393. doi: 10.3390/cimb48040393 (PMC13115264; doi:10.3390/cimb48040393)

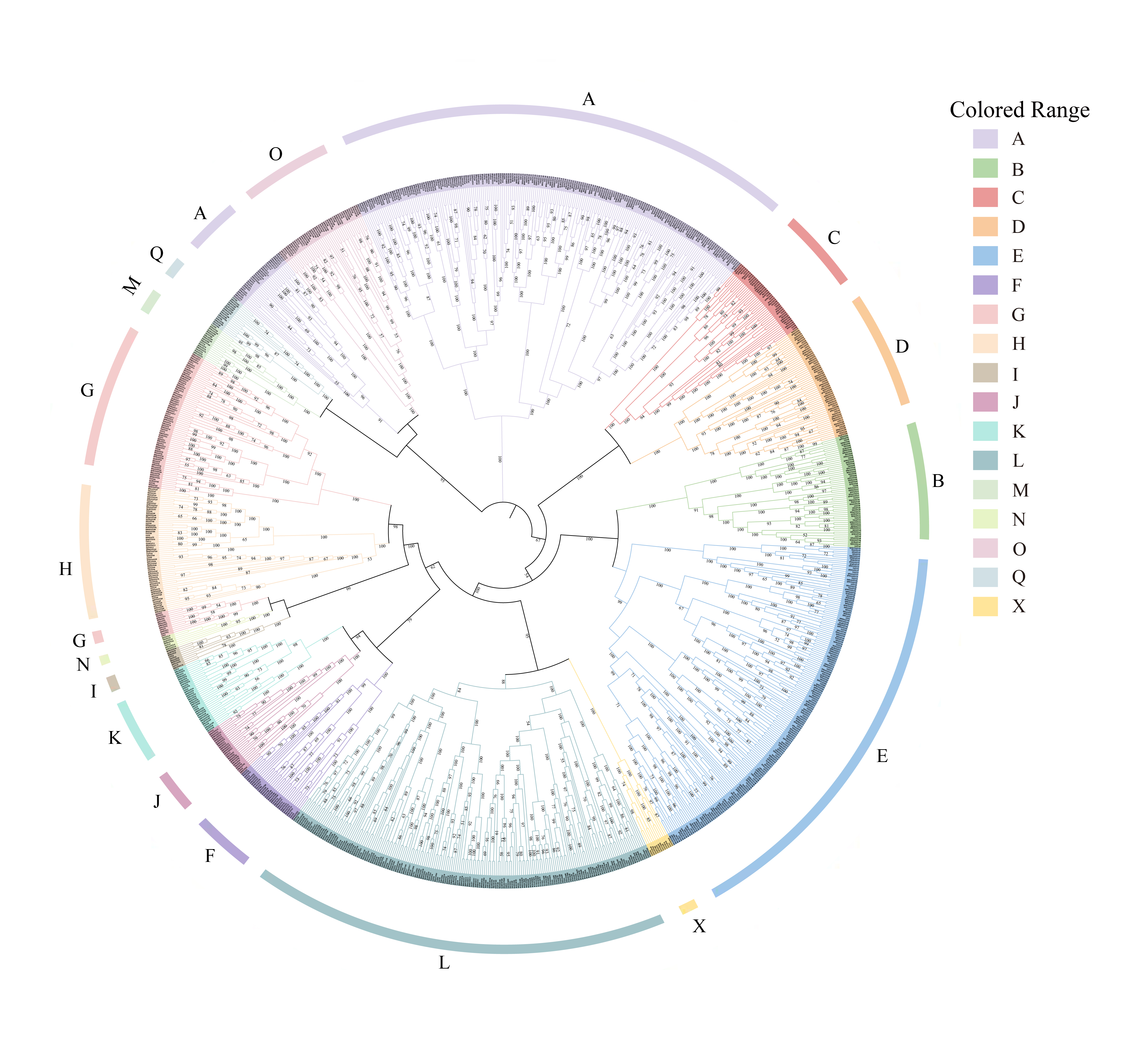

Supplement: Supplementary file 1 [file cimb-48-00393-s001.zip › cimb-4218483-supplementary-xml/Figure in Appendix/Figure 1 shows the phylogenetic tree.jpg]
